# LC-HRMS data as a result of untargeted metabolomic profiling of human cerebrospinal fluid

**DOI:** 10.1016/j.dib.2018.10.113

**Published:** 2018-10-27

**Authors:** Florence Mehl, Héctor Gallart-Ayala, Ioana Konz, Tony Teav, Aikaterini Oikonomidi, Gwendoline Peyratout, Vera van der Velpen, Julius Popp, Julijana Ivanisevic

**Affiliations:** a Metabolomics Platform, Faculty of Biology and Medicine, University of Lausanne, Switzerland; b Old Age Psychiatry, Department of Psychiatry, Lausanne University Hospital, Switzerland; c Geriatric Psychiatry, Department of Mental Health and Psychiatry, Geneva University Hospitals, Switzerland

## Abstract

Cerebrospinal fluid (CSF) is a key body fluid that maintains the homeostasis in central nervous system (CNS). As a biofluid whose content reflects the brain metabolic activity, the CSF is analyzed in the context of neurological diseases and is rarely collected from healthy subjects. For this reason, the metabolite variation associated with general phenotypic characteristics such as gender and age have hardly ever been studied. Here we present the hydrophilic interaction liquid chromatography-high resolution mass spectrometry (HILIC-HRMS) data as a result of untargeted metabolomics analysis of a cohort of elderly cognitively healthy volunteers (*n* = 32). 146 unambiguously identified water soluble metabolites (using accurate mass, retention time and MS/MS matching against spectral libraries) were measured and their abundances across all the subjects depending on their gender are provided in this article. Data tables are available at https://data.mendeley.com/datasets/c73xtsd4s5/1. it's published on mendeley, the DOI is DOI:10.17632/c73xtsd4s5.1. The data presented in this article are related to the research article entitled “A global HILIC-MS approach to measure polar human cerebrospinal fluid metabolome: Exploring gender-associated variation in a cohort of elderly cognitively healthy subjects” (Gallart-Ayala et al., 2018, In press).

**Specifications table**

**Table:** 

Subject area	Clinical study
More specific subject area	Metabolomics
Type of data	XCMS preprocessed and signal drift corrected LC–MS data
How data was acquired	Global-untargeted metabolite profiling was performed using HILIC coupled to high-resolution mass spectrometry (HRMS) operating in both positive and negative (ESI+ and ESI-) mode on 6550 iFunnel Q-TOF mass spectrometer interfaced with 1290 UHPLC system (Agilent Technologies). Data were acquired in the full *m/z* range 50–1200 in both modes.
Data format	Tables in.txt format
Experimental factors	Cerebrospinal fluid samples were collected using lumbar puncture from cognitively healthy, elderly volunteers (*n* = 32, with no history or symptoms of relevant psychiatric or neurologic disease, no cognitive impairment, and without cerebral Alzheimer׳s disease pathology as indicated by CSF biomarkers). Clinical characteristics of the cohort are provided below in Experimental design section.
Experimental features	Cerebrospinal fluid samples were extracted by the addition of ice-cold MeOH:ACN (1:1). CSF extracts were analyzed with ESI-LS-MS system and processed using ProFinder (Agilent Technologies) and XCMS software. For the quality control (QC), pooled QC samples (representative of the entire sample set) were analyzed periodically (every 7 samples) throughout the run.
Data source location	Metabolomics Platform, Faculty of Biology and Medicine, University of Lausanne, Lausanne, Switzerland
Data accessibility	Data tables are available at https://data.mendeley.com/datasets/c73xtsd4s5/1. it's published on mendeley, the DOI is DOI:10.17632/c73xtsd4s5.1

**Value of the data**

• The dataset consists of the LC-HRMS metabolomics data as a result of untargeted analyses of cerebrospinal fluid samples from a cohort of elderly cognitively healthy human subjects (*n* = 32).
• In this paper [1], metabolites were unambiguously identified by AMRT in-house database matching and MS/MS spectra matching against publically available spectral libraries (METLIN & mzCloud).
• The identified metabolites (*n* = 146) are implicated in multiple metabolic pathways including glycolysis and TCA cycle, purine and pyrimidine, amino acid and fatty acid metabolism.
• The acquired data allowed for the exploration inter-individual variability and the discovery of gender-associated differences.

## 1 Data

Data tables are available at https://data.mendeley.com/datasets/c73xtsd4s5/1. it's published on mendeley, the DOI is DOI:10.17632/c73xtsd4s5.1. The dataset contains the XCMS processed and signal drift corrected data. Data table is reported in.txt format and contains the information (i.e. peak areas) about all detected metabolite features across analyzed samples and pooled quality control sample. Metabolite features are assigned by the couple of specific RT and accurate *m*/*z* ratio information and annotated with CAMERA for isotopes. Peaks were detected and integrated using XCMS software (parameters are specified in Materials and Methods). Metabolites abundances were corrected for signal drift effect by fitting a locally quadratic (loess) regression model to the QC values (as described in Materials and Methods below). A table of all identified metabolites derived from this dataset is available as supplementary table in the research article entitled “A global HILIC-MS approach to measure polar human cerebrospinal fluid metabolome: Exploring gender-associated variation in a cohort of elderly cognitively healthy subjects” [1]. These data were log transformed prior to univariate statistical analysis to explore gender-associated differences.

## 2 Experimental design, materials and methods

### 2.1 Study population and CSF sample collection

Cerebrospinal fluid samples were collected using lumbar puncture from cognitively healthy (with no history or symptoms of relevant psychiatric or neurologic disease, and no cognitive impairment) volunteers (*n* = 32) at the Department of Psychiatry and the Department of Clinical Neurosciences, University Hospital in Lausanne, Switzerland (CHUV). The participants have been selected among cognitively healthy, community dwelling volunteers participating on an observational cohort study on cognitive aging and Alzheimer׳s disease (AD) [2]. They were recruited by announcements and word of mouth. All participants had a comprehensive medical, neuropsychological, and psychosocial evaluation to exclude cognitive impairment, as well as brain MRI or CT scans, and venous and lumbar punctures. As a significant percentage of elderly people with normal cognition may have cerebral AD pathology, only participants with a non-AD CSF biomarker profile have been included in this study [2]. The study was approved by the cantonal ethics committee (Vaud) and informed consent was obtained from all participants [3].

Lumbar punctures were performed between 8 and 10 a.m. after an overnight fast. For lumbar puncture, a standardized technique with a 22-gauge “atraumatic” spinal needle and the subject in a sitting or lying position was applied [4]. A volume of 10–12 ml of CSF was collected in polypropylene tubes. CSF samples were centrifuged, frozen in aliquots, and stored at −80 °C before further use (Table 1).

**Table 1:** Clinical characteristics of the cohort studied defining median and inter-quartile range.

Characteristic	
Gender (male:female)	12:20
Age (median, IQR), years	65.0 (62.5,70.5)
BMI (median, IQR)	23.8 (21.8,26.1)
Smokers (non:past:active)	12:16:4
Cholesterol (median, IQR), mmol L^−1^	6.1 (5.5,7.0)^a^
Glucose (median, IQR), mmol L^−1^	5.6 (5.1,6.0)^a^
Creatinine (median, IQR), mmol L^−1^	76.0 (61.0,89.3)

a Data not available for 1 subject.

### 2.2 CSF metabolite extraction

Cerebrospinal fluid samples (100 μL) were extracted by the addition of 400 μL of ice-cold MeOH:ACN (1:1, v/v) to maintain MeOH:ACN:H_2_O (2:2:1, v/v) ratio. The samples were then vortexed for 30 s, incubated for 1 h at −20 °C, and finally centrifuged at 13,000 rpm at 4 °C for 15 min. The resulting supernatant was evaporated to dryness using a speedVac. The dry extracts were then reconstituted in 100 μL of H_2_O:MeOH:ACN (2:1:1, v/v), sonicated for 1 min and centrifuged for 10 min at 13,000 rpm at 4 °C to remove the insoluble debris. The supernatants were transferred to HPLC vials and stored at −80 °C prior to LC-MS analysis.

### 2.3 Untargeted LC–HRMS analysis

Global-untargeted metabolite profiling was performed by HILIC chromatography coupled to high-resolution mass spectrometry (HRMS) operating in both positive and negative (ESI+ and ESI-) mode on 6550 iFunnel Q-TOF mass spectrometer interfaced with 1290 UHPLC system (Agilent Technologies). Samples were analyzed using two chromatographic separations: i) BEH Amide, 1.7 μm, 100 mm × 2.1 mm I.D. column (Waters, Massachusetts, US) in positive ionization mode and, ii) SeQuant® ZIC-pHILIC, 5 μm, 100 mm × 2.1 mm I.D. column (Merck, Darmstadt, Germany) with a SeQuant® ZIC-pHILIC, 5 μm, 20 mm × 2.1 mm I.D. guard column (Merck, Darmstadt, Germany) in negative ionization mode. The column temperature was maintained constant at 25 °C and 30 °C in positive and negative ionization mode, respectively. The mobile phase was composed of A = 20 mM ammonium formate and 0.1% formic acid in water (pH 3.8) and B = 0.1% formic acid in 100% ACN for positive mode and A=20 mM ammonium acetate and 20 mM ammonium hydroxide in water (pH 9.3) and B = 100% ACN for negative mode. In positive mode, the linear elution gradient from 95% B (0–1.5 min) to 45% B (17–19 min) was applied. The initial gradient conditions were restored within one minute and a 5-min post-run re-equilibration was applied to maintain the system reproducibility. In negative mode, the linear step-wise elution gradient from 90% B (0–1.5 min) to 50% B (8–11 min) to 45% B (12–15 min) was applied. The initial gradient conditions were restored within one minute and a 9-min post-run equilibration was applied to maintain the system reproducibility. The flow rates were 400 μL min^−^^1^ and 300 μL min^−^^1^ in positive and negative ionization mode, respectively. In both cases, the sample injection volume was 2 µl. ESI source conditions were set as follows: dry gas temperature 290 °C and flow 14 L min^−1^, fragmentor voltage 380 V, sheath gas temperature 350 °C and flow 12 L min^−1^, nozzle voltage 0 V, and capillary voltage +2000 V in positive mode and −2000 V in negative. The instrument was set to acquire over the full *m/z* range 50–1200 in both modes, with the MS acquisition rate of 2 spectra/s. In addition, targeted MS/MS analysis was performed using the inclusion list of ions of interest, in narrow isolation width (~1.3 *m/z*), with a MS acquisition rate of 500 ms and a MS/MS acquisition rate of 500 ms at a collision energy (CE) of 20 V. For the quality control (QC), pooled QC samples (representative of the entire sample set) were analyzed periodically (every 7 samples) throughout the overall analytical run to assess the analytical variability and correct for the potential signal intensity drift inherent to LC-MS technique [5].

### 2.4 Data processing

Raw LC-MS data were converted to mzXML files (using ProteoWizard MS Convert) and uploaded to XCMS plus software (stand-alone server hosted within bioinformatics facility at UNIL) for data processing including peak detection, retention time correction, profile alignment, and isotope annotation [6], [7], [8]. Data were processed as a multi-group experiment and the parameter settings were as follows: centWave algorithm for feature detection (Δ*m/z* = 20 ppm, minimum peak width = 5 s and maximum peak width = 30 s, S/N threshold = 10, mzdiff=0.01, integration method = 1, prefilter peaks = 3, prefilter intensity = 10000, noise filter = 5000); obiwarp settings for retention time correction (profStep = 1); and parameters for chromatogram alignment, including mzwid = 0.015, minfrac = 0.5 and bw = 5 [9].

### 2.5 Signal intensity drift correction and statistical analysis

The data table containing the abundances or peak areas of all detected and identified metabolites across all samples (CSF extracts from 32 individuals) was imported to Workflow4Metabolomics [10], [11] where the peak intensities were corrected for signal drift effect by fitting a locally quadratic (loess) regression model to the QC values [5], [12]. The α parameter controlling the smoothing was set to 1 to avoid over fitting [11].
